# Biomarkers of PUFA and cardiovascular risk factors and events in healthy Asian populations: a systematic review

**DOI:** 10.1017/S0007114524002708

**Published:** 2024-12-14

**Authors:** Yu Qi Lee, Kok Hsien Tan, Mary F.-F. Chong

**Affiliations:** 1 Saw Swee Hock School of Public Health, National University of Singapore, Singapore; 2 Institute for Human Development and Potential, Agency for Science, Technology, and Research, Singapore

**Keywords:** N-3 PUFA, N-6 PUFA, Biomarkers, Cardiovascular, Asian

## Abstract

The associations between circulating PUFA and cardiovascular risk factors and events in healthy Asian populations have been less examined robustly compared with Western populations. This systematic review aimed to summarise current evidence on the associations between *n*-3 and *n*-6 PUFA biomarkers and cardiovascular risk factors and events in healthy Asian populations. Four databases were searched for observational studies from 2010 until 2024. Twenty-three studies were eligible, which covered six Asian countries and included events (*n* 7), traditional risk factors such as blood pressure and lipids (*n* 4), physical signs such as arterial stiffness (*n* 4), non-traditional lipid markers (*n* 1), markers of inflammation (*n* 4), markers of thrombosis (*n* 2) and non-invasive imaging-based markers (*n* 5). Biological sample types included plasma (*n* 6), serum (*n* 14) and erythrocyte (*n* 3). Higher circulating total *n*-3 PUFA appeared to be associated with lower hypertension risk and specifically EPA and DHA to be associated with lower myocardial infarction risk, reduction in TAG and inflammation. Higher circulating linoleic acid was associated with improved lipid profiles and lower inflammation. Limited evidence led to inconclusive associations between circulating *n*-6 PUFA biomarkers and CVD events and blood pressure. No consistent associations with arterial stiffness, obesity, thrombosis and imaging-based biomarkers were observed for circulating PUFA biomarkers in Asian populations. Limited studies exist for each outcome; hence, results should be interpreted with caution. More high-quality and prospective studies in Asian populations are warranted. Several recommendations such as sample size justification and reporting of non-respondents rate are proposed for future studies.

CVD is the leading cause of deaths globally and in Asia in 2019^(1)^. About one-third of CVD deaths, or 12 % of total deaths, were attributable to dietary risks, which formed the leading behavioural risk factor for CVD deaths^(2)^. Among various dietary components, PUFA, especially *n*-3 and *n*-6, have gained substantial attention due to their potential health benefits.

Dietary intake or supplementation of *n*-3 PUFA has been shown to have favourable effects on lipid profiles^(3)^, inflammation^(3,4)^, blood pressure^(3,5,6)^ and thrombosis^(7)^, all of which are well-established risk factors for CVD. Long-term prospective cohort studies consistently demonstrated an inverse association between fish and *n*-3 PUFA intake and the risk of developing CVD, especially CHD and myocardial infarction (MI) and cardiovascular mortality in the general population^(8,9)^. However, a recent Cochrane review found that *n*-3 PUFA supplementation with capsules has little or no effect on combined cardiovascular events (including fatal and non-fatal MI, angina, stroke, heart failure and peripheral arterial disease) or cardiac death^(10)^. In line with the findings from observational studies, a meta-analysis of prospective studies in Western and Asian populations indicated that higher levels of *n*-3 PUFA biomarkers were associated with a significantly reduced risk of total CVD, CHD and total mortality^(11)^. In this meta-analysis, only four studies examining CVD-related outcomes were conducted in Asia^(11)^. Associations between *n*-3 PUFA biomarkers and cardiovascular risk factors and events specifically in healthy Asian populations have been less studied.

A recent meta-analysis found the associations between circulating or dietary *n*-3 PUFA and metabolic syndrome risk to be different between Asian and American/European populations^(12)^. Asian participants with a high *n*-3 PUFA level had a lower metabolic syndrome risk, while no such associations were observed in American/European participants^(12)^. Dietary patterns vary between Western and Asian populations^(13)^ and evidence from trans-ethnic studies showed genetic heterogeneities involving fatty acid metabolism^(14)^ and type 2 diabetes susceptibility^(15,16)^ between Caucasians and Asians. These factors may result in differences in the association between biomarkers of PUFA and cardiovascular risk factors and events. Thus, it is important to examine the associations between biomarkers of PUFA and cardiovascular risk factors and events in Western and Asian populations separately to account for the differential effects in various populations.

The role of *n*-6 PUFA in cardiovascular health remains controversial. A Cochrane review found that increasing intake of *n*-6 PUFA from food or supplements may reduce serum total cholesterol (TC) but not other blood fat fractions or adiposity^(17)^. Also, *n*-6 PUFA intake was not associated with any CVD events and mortality^(17,18)^. However, a meta-analysis reported an association between a higher level of linoleic acid from adipose tissue and blood and a lower risk of CVD and CVD mortality^(19)^. The conflicting results might be related to variations in the method of measurement of PUFA (dietary *v*. biomarker) and measurement errors of dietary assessments, as well as the bioavailability of these fatty acids^(20)^. Like the above regarding *n*-3 PUFA biomarkers, associations between *n*-6 PUFA biomarkers and cardiovascular risk factors and events specifically in healthy Asian populations have been less studied.

Dietary assessment by 24-h dietary recalls, food records or FFQ could be subjected to reporting bias and measurement errors. Biomarkers of PUFA provide a more objective and reliable assessment of PUFA intake because they are free of reporting bias and other measurement errors intrinsic to questionnaire-based assessments. Therefore, biomarkers of *n*-3 or *n*-6 PUFA are valuable when evaluating the associations between the intake of these PUFA and disease risk^(21)^. Additionally, biomarker-based investigations allow direct quantification of individual fatty acids, which may have different effects on clinical outcomes or disease risk factors^(22)^.

The associations between biomarkers of *n*-3 and *n*-6 PUFA and cardiovascular risk factors and events in healthy Asian populations have been less examined robustly compared with Western populations and remain uncertain. To our knowledge, there has been no systematic review summarising the existing evidence from observational studies specifically in Asian populations. Therefore, this study aimed to systematically review the associations between biomarkers of *n*-3 and *n*-6 PUFA and cardiovascular risk factors and events in healthy Asian populations, to discuss the heterogeneity between studies and to identify the existing gaps in the literature.

## Methods

The protocol for this systematic review was registered in the International Prospective Register of Systematic Reviews (PROSPERO) as CRD42022354471^(23)^ and followed the guidelines outlined in the Preferred Reporting Items for Systematic Reviews and Meta-Analyses (PRISMA) statement^(24)^.

### Search strategy and selection criteria

Relevant studies published from January 2010 until August 2024 were searched using databases PubMed, Embase, Web of Science and Cochrane Library. We intend to include only recent evidence. The keywords used for the search were ‘omega-3 fatty acid’ OR ‘*n*-3’ OR ‘omega-6 fatty acid’ OR ‘*n*-6’ OR ‘polyunsaturated fatty acid’ or ‘PUFA’ OR ‘omega’ AND ‘Asia’. The Boolean operator ‘AND’ was used between groups and ‘OR’ within groups. The search strategy incorporated systematic reviews and meta-analyses to enable the exploration of reference lists within these reviews for any additional relevant articles.

Published original studies meeting the following criteria were included: (1) observational studies (cross-sectional, cohort, nested case–control and case–control) that examined *n*-3 and/or *n*-6 PUFA in circulating whole blood, serum, plasma, erythrocyte or adipose tissue; (2) studies that examined the relationship between PUFA biomarkers and any health outcomes or risk factors including (but not limited to) inflammation, non-communicable diseases, metabolic syndrome, type 2 diabetes, all types of CVD, all types of cancer, all-cause mortality or cause-specific mortality; (3) studies conducted in Asian (East Asian, Southeast Asian and South Asian) populations; and (4) any healthy populations, which was defined as individuals without any chronic diseases. No age restriction was given. The following studies were excluded: (1) studies where subjects were pregnant/nursing women, migrant populations or with existing chronic diseases; (2) studies that reported only dietary PUFA intake or PUFA supplementation; (3) animal studies; (4) studies that were case studies or randomised clinical trials, (5) non-original articles such as reviews, commentary or letters; and (6) non-English publications. The inclusion of studies was first assessed based on their title and abstract. For articles that met the inclusion criteria, the articles were then retrieved and reviewed, and relevant data were extracted. All stages in this process were carried out by two independent reviewers (YQ, KH or Jason). Any disagreements were resolved through discussion to reach mutual consensus and/or consultation with a third independent reviewer. This paper will only focus on CVD-related risk factors and events as the health outcome of interest. The other health outcomes will be reported separately.

### Data extraction

Two authors (YQ and KH) independently extracted and adjudicated data on pre-specified data collection tables and cross-checked them for accuracy and consistency. Any discrepancies were resolved by consensus. The following data were extracted: publication details (publication year and country), study and participant characteristics (study type, aim, sample size, age and sex, duration of follow-up (for prospective studies), biological sample type and method of measurement, PUFA type and health outcome. Due to both heterogeneity in reporting and the limited number of studies reporting data for the same combinations of PUFA biomarkers (exposures) and cardiovascular risk factors and events (outcomes), a meta-analysis was deemed unsuitable, and studies were summarised narratively.

### Quality assessment

Quality assessment of the included studies was performed by two researchers (YQ and KH) independently using the Newcastle–Ottawa Scale (NOS)^(25)^. The NOS assigns a maximum of nine stars for cohort and case–control studies, evaluating three domains: (1) selection of study groups (up to four stars), (2) comparability of groups (up to two stars) and (3) ascertainment of outcome (for cohort studies) or exposure (for case–control studies) (up to three stars). An adapted NOS was used for cross-sectional studies^(26)^. The NOS assigns a maximum of ten stars, evaluating three domains: (1) selection of study groups (up to five stars), (2) comparability of groups (up to two stars) and (3) ascertainment of outcome (up to three stars). Any disagreements in the quality assessment were resolved through discussion to reach a mutual consensus and/or consultation with a third independent reviewer. No studies were excluded based on quality ratings.

## Results

### Search results

**Fig. 1.**
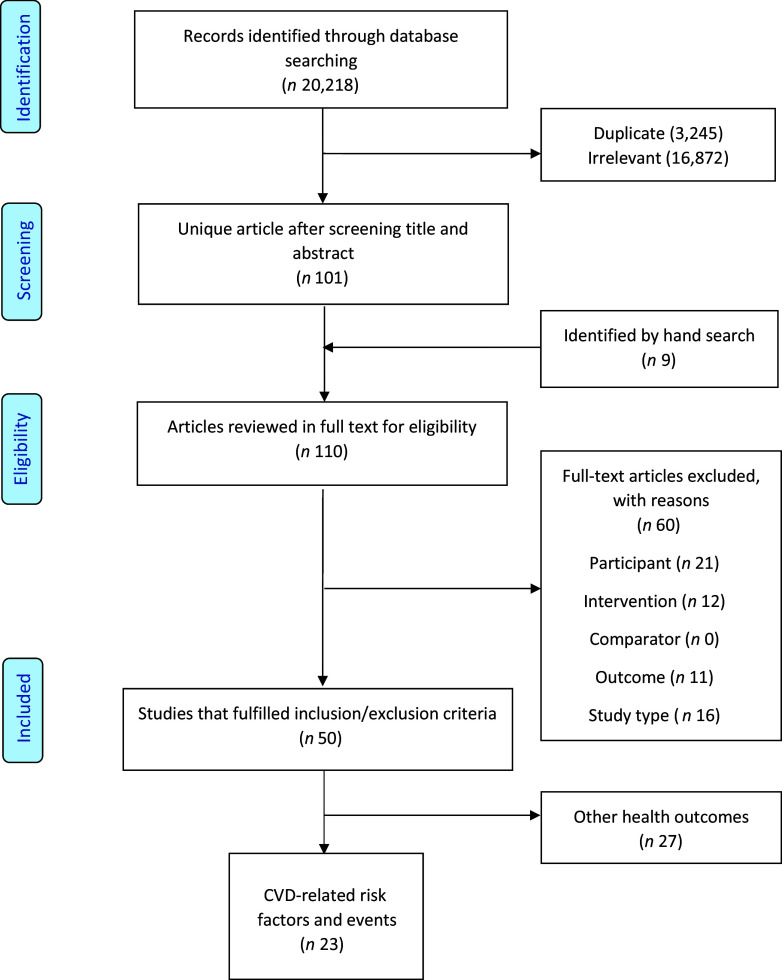
Preferred Reporting Items for Systematic Reviews and Meta-analysis (PRISMA) flow diagram of the study selection process.

A total of 20 218 articles were identified in our initial search through the databases, of which 3245 were duplicates and 16 872 were excluded based on a review of titles and abstracts. An additional nine articles were identified by hand searching. Full text of the remaining articles was retrieved, leading to the exclusion of another sixty because they do not meet the PICOS criteria: population (*n* 21), intervention (*n* 12), comparator (*n* 0), outcome (*n* 11) and study type (*n* 16). Of the remaining fifty studies, twenty-three studies focused on CVD-related risk factors and events and were included in this present paper. A flow diagram of the search result is illustrated in Fig. 1.

### Study characteristics

Of the twenty-three studies that looked at CVD-related outcomes, study types included cross-sectional (*n* 16), prospective nested case–control and case–control (*n* 5) and prospective cohorts (*n* 2^1^) (one study consisted of both a cross-sectional and prospective cohort component)) with follow-up range of 3–9·6 years; countries included Japan (*n* 13), China (*n* 4), Singapore (*n* 3), Korea (*n* 2), India (*n* 1) and Taiwan (*n* 1); sample size ranged from 100 to 2206 for cross-sectional studies, 146–1488 for case–control studies and 1477–1833 for cohort studies; and age ranged from 39 to 85. PUFA examined included total *n*-3, EPA, DHA, DPA, *α*-linolenic acid (ALA), total *n*-6, arachidonic acid, linoleic acid and dihomo-*γ*-linolenic acid (DGLA). Biological sample types included plasma (*n* 6), serum(*n* 14) and erythrocyte(*n* 3).

The categorisation of outcomes related to CVD^(27)^ found in this systematic review is shown in Table 1. CVD events included selected composite CVD events (non-fatal MI, fatal CHD, hospitalisation due to percutaneous coronary intervention or coronary bypass surgery and stroke)^(28)^, coronary artery disease^(29)^, total stroke^(30)^, ischaemic stroke^(30)^, intracerebral haemorrhage^(30)^, atrial fibrillation^(31)^, MI^(32–34)^ and cardiac death^(34)^. Traditional risk factors included blood pressure (systolic blood pressure and diastolic blood pressure)^(35–37)^, lipids panel (TC, TAG, HDL-cholesterol, LDL-cholesterol)^(37,38)^, fasting blood glucose, fasting serum insulin and homeostatic model assessment for insulin resistance^(37)^. Physical signs included aortic stiffness^(39)^, arterial stiffness/wave reflection^(40)^, BMI^(38)^, fat mass and waist circumference^(37)^. Non-traditional lipid markers included VLDL-cholesterol, LDL-cholesterol and HDL-cholesterol subclasses^(41)^. Inflammatory markers included C-reactive protein (CRP)^(40,42,43)^ and proinflammatory cytokines^(44)^. Markers of haemostasis and thrombosis included plasma fibrinogen^(45)^ and plasma plasminogen activator inhibitor-1 (PAI-1)^(46)^. Non-invasive imaging-based markers included coronary artery calcification (CAC) score^(47)^, CAC density score^(47)^, aortic calcification score^(48)^ and carotid intima media thickness (IMT)^(49,50)^. The main characteristics of these studies are summarised in Table 2.

**Table 1.** Categorisation of CVD outcomes, comprising events, risk factors and biomarkers, adapted from (27)

Categories	Examples
Events	CVD events, total stroke, ischaemic stroke, intracerebral haemorrhage, myocardial infarction, atrial fibrillation, CAD, cardiac death
Traditional risk factors	Blood pressure, dyslipidaemia, fasting blood glucose, fasting serum insulin, HOMA-IR
Physical signs	Obesity, BMI, ankle-brachial index
Plasma-based biomarkers • Non-traditional lipid markers • Markers of inflammation • Markers of haemostasis and thrombosis	• Lipoprotein particle size, particle density • CRP, proinflammatory cytokines • PAI-1, fibrinogen
Imaging-based biomarkers	Carotid IMT, coronary calcification

CAD, coronary artery disease; HOMA-IR, homeostatic model assessment of insulin resistance; CRP, C-reactive protein; PAI-1, plasminogen activator inhibitor-1; IMT, intima media thickness.

**Table 2.** Characteristics and results of studies

Health outcome	Author, year	Study design, country	Sample size, sex, age (years)	Biological sample	Total *n*-3	EPA	DHA	DPA	ALA	Total *n*-6	AA	LA	DGLA	Significant results
CVD events														
CVD events	Chien KL *et al.*, 2013 [28]	Prospective cohort, Taiwan	1833, male and female, 60·6 ± 10·5	Plasma	–	↔	↔	–	–	–	–	–	–	**–**
CAD (MI, angina pectoris, cardiac death)	Chei CL *et al.*, 2018 [29]	Prospective nested case–control, Japan	152 cases, 456 controls, male and female, 40–85	Serum	↔	↔	↔	↔	↔	↓	↔	↓	–	Total *n*-6: OR 0·66 (95 % CI 0·52, 0·84), *P* < 0·001 LA: OR 0·67 (95 % CI 0·53, 0·85), *P* < 0·001
Total stroke, ischaemic stroke, intracerebral haemorrhage	Sun L *et al.*, 2022 [30]	Prospective cohort, China	412 incident stroke cases, 7747 none-stroke, male and female, 58·1 (sd 9·9) years	Erythrocyte	↔	↓ (Total stroke, ischaemic stroke)	↔	↔	↔	↔	↔	↓ (Ischaemic stroke) ↑ (Intracerebral haemorrhage)	↑ (Total stroke, ischaemic stroke)	EPA: Inverted–*U* shape curve associations with total stroke (*P*-nonlinear = 0·008) and ischaemic stroke (*P*-nonlinear = 0·002) with a threshold at EPA level of 0·70 % LA: Ischaemic stroke HR 0·69 (95 % CI 0·53, 0·90), *P*-trend = 0·027 Intracerebral haemorrhage HR 2·33 (95 % CI 1·41, 3·82), *P*-trend = 0·007 DGLA: Total stroke HR 1·51 (95 % CI 1·23, 1·84), *P*-trend = 0·001 Ischaemic stroke HR 1·64 (95 % CI 1·32, 2·04), *P*-trend = 0·001
AF	Tomita T *et al.*, 2013 [31]	Case-control, Japan	110 patients with AF and 36 healthy volunteers, male and female, ∼65–66	Serum	–	↑	↔	–	–	–	↔	–	–	EPA: OR 1·02 (95 % CI 1·00, 1·04), *P* = 0·03
Risk of MI, cardiac death	Hamazaki K *et al.*, 2018 [34]	Prospective nested case–control, Japan	209 cases and 418 controls, male and female, ∼57	Plasma	↓ (Cardiac death) ↔ (MI)	↔	↔	↔	–	–	–	–	–	Q4 OR 0·08 (95 % CI 0·01, 0·88), *P*-trend = 0·04
Risk of MI	Sun Y *et al.*, 2016 [32]	Prospective nested case–control, Singapore	727 incident acute MI cases and 727 matched controls, male and female, 47–83	Plasma	↓(EPA + DHA)	↓	↓	–	–	–	↑	↔	–	Total *n*-3: OR 0·67 (95 % CI 0·53, 0·84), *P* < 0·001 EPA: OR 0·74 (95 % CI 0·62, 0·88), *P* = 0·001 DHA: OR 0·75 (95 % CI 0·60, 0·93), *P* = 0·008 AA: OR 1·25 (95 % CI 1·03, 1·52), *P* = 0·02
Risk of MI	Sun Y *et al.*, 2016 [33]	Prospective nested case–control, Singapore	744 incident acute MI cases and 744 matched controls, male and female, 47–83	Plasma	↓(EPA + DHA)	↓	↓	–	↔	–	–	–	–	Total *n*-3: OR 0·62 (95 % CI 0·41, 0·94), *P* = 0·03 EPA: OR 0·68 (95 % CI 0·46, 1·00), *P* = 0·02 DHA: OR 0·59 (95 % CI 0·38, 0·90), *P* = 0·01
Traditional risk factors														
Blood pressure	Zeng FF *et al.*, 2014 [36]	Cross-sectional, China	1834, male and female, 57 ± 5	Erythrocyte	↓	–	–	–	–	↓	–	–	–	Total *n*-3: *P*-trend SBP 0·002, DBP < 0·001 Total *n*-6: *P*-trend SBP 0·001, DBP < 0·001
		Prospective cohort, China	1477, male and female, 57 ± 5		↓	↓	↓	↓	↔	↔	–	–	–	Total *n*-3: *P*-trend SBP 0·008, DBP 0·029 EPA: *P*-trend SBP 0·004, DBP < 0·001 DHA: *P*-trend SBP 0·005, DBP 0·017 DPA: *P*-trend SBP 0·004, DBP 0·044
Blood pressure	Huang T *et al.*, 2012 [35]	Cross-sectional, China	1154, male and female, ∼44–48	Plasma	↓	↔	↔	↔	↔	↔	↔	↔	–	Total *n*-3: OR 0·43 (95 % CI 0·29, 1·19), *P* = 0·046
Blood pressure	Bi X *et al.*, 2019 [37]	Cross-sectional, Singapore	172, male and female, ∼39	Plasma	↔	–	–	–	–	↔	–	–	–	–
Cholesterol, TAG, HDL-cholesterol, LDL-cholesterol	Gupta R *et al.*, 2013 [38]	Cross-sectional, India	176, male and female, 47·23 ± 6·00	Serum	↓(EPA + DHA) (TC, TC:HDL-cholesterol ratio, TAG)	–	–	–	–	–	–	↓(TAG)	–	Total *n*-3: TC, TC:HDL-cholesterol ratio, TAG *P* < 0·05 LA: TAG *P* < 0·05
Cholesterol, TAG, HDL-cholesterol, LDL-cholesterol	Bi X *et al.*, 2019 [37]	Cross-sectional, Singapore	172, male and female, ∼39	Plasma	↓(TC)	↓(TAG, TC)	–	–	–	↓(TAG, TC, LDL-cholesterol) ↑(HDL-cholesterol)	↓(LDL-cholesterol)	↓(TAG, TC, LDL-cholesterol) ↑(HDL-cholesterol)	–	Total *n*-3: TC: β –0·19 (*P* < 0·05) EPA: TAG: *r* = –0·18 (*P* = 0·020) TC: *r* = –0·21 (*P* = 0·007) Total *n*-6: TAG: β –0·25 (*P* < 0·005) TC: β –0·22 (*P* < 0·05) LDL-cholesterol: β –0·19 (*P* < 0·05) HDL-cholesterol: β 0·21 (*P* < 0·05) AA: LDL-cholesterol: *r* = –0·16 (*P* = 0·035) LA: TAG: *r* = –0·16 (*P* = 0·040) TC: *r* = –0·17 (*P* = 0·025) LDL-cholesterol: *r* = –0·23 (*P* = 0·003) HDL-cholesterol: *r* = 0·27 (*P* < 0·001)
FG, FSI, HOMA-IR	Bi X *et al.*, 2019 [37]	Cross-sectional, Singapore	172, male and female, ∼39	Plasma	↔ (FG, FSI, HOMA-IR)	–	–	–	–	↔ (FG, FSI, HOMA-IR)	–	–	–	–
Physical signs														
Aortic stiffness	Sekikawa A *et al.*, 2013 [39]	Cross-sectional, Korean	299, male, 40–49	Serum	↓ (EPA + DHA + DPA)	↓	↔	–	–	–	–	–	–	Total *n*-3: β –0·132 (*P* = 0·02) EPA: β –0·149 (*P* = 0·008)
Arterial stiffness/wave reflection	Tomiyama H *et al.*, 2011 [40]	Cross-sectional, Japan	2206, male, 44 ± 9	Serum	↔	↔	↔	–	–	–	↑	–	↑	AA: β 0·02 (95 % CI 0·01, 0·03), *P* < 0·01 DGLA: β 1·02 (95 % CI 0·35, 1·70), *P* < 0·01
BMI	Gupta R *et al.*, 2013 [38]	Cross-sectional, India	176, male and female, 47·23 ± 6·00	Serum	–	–	–	–	↓	–	–	–	–	ALA: *P* < 0·05
Fat mass, WC	Bi X *et al.*, 2019 [37]	Cross-sectional, Singapore	172, male and female, ∼39	Plasma	↔ (Fat mass, WC)	–	–	–	–	↓(WC) ↔ (Fat mass)	–	–	–	Total *n*-6: WC: β –0·18 (*P* < 0·05)
Non-traditional lipid markers														
Lipoprotein subclass VLDL-cholesterol, LDL-cholesterol, HDL-cholesterol	Choo J *et al.*, 2010 [41]	Cross-sectional, Japan, Korea,	303 Japanese 298 Koreans, males, 40–49	Serum	–	–	–	–	–	VLDL-cholesterol (total, large, medium, size) J↓ K↓ VLDL-cholesterol (small) J↑ K↑	VLDL-cholesterol (total, large, medium, size) J↓ K↓ VLDL-cholesterol (small) J↔ K↔	VLDL-cholesterol (total) J↓ K↔ VLDL-cholesterol (large, medium, size) J↓ K↓ VLDL-cholesterol (small) J↑ K↑	–	For particle size Total *n*-6: J: β –0·90 (*P* < 0·001) K: β –1·34 (*P* < 0·001) AA: J: β –1·57 (*P* < 0·001) K: β –2·29 (*P* < 0·001) LA: J: β –0·74 (*P* < 0·001) K: β –1·30 (*P* < 0·001)
										LDL-cholesterol (total, IDL, small) J↓ K↓ LDL-cholesterol (large, size) J↑ K↑	LDL-cholesterol (total) J↓ K↔ LDL-cholesterol (IDL) J↔ K↔ LDL-cholesterol (small) J↓ K↓ LDL-cholesterol (large, size) J↑ K↑	LDL-cholesterol (total, IDL, small) J↓ K↓ LDL-cholesterol (large) J↑ K↑ LDL-cholesterol (size) J↑ K↑		For total LDL-cholesterol Total *n*-6: J: β –21·44 (*P* < 0·001) K: β –22·92 (*P* < 0·001) AA: J: β –37·34 (*P* < 0·05) LA: J: β –19·05 (*P* < 0·001) K: β –27·47 (*P* < 0·01)
										HDL-cholesterol (total, medium, small) J↓ K↓ HDL-cholesterol (large, size) J↑ K↑	HDL-cholesterol (total, medium, small) J↔ K↔ HDL-cholesterol (large, size) J↑ K↑	HDL-cholesterol (total, medium, small) J↓ K↓ HDL-cholesterol (large, size) J↑ K↑		For particle size Total *n*-6: J: β 0·04 (*P* < 0·001) K: β 0·03 (*P* < 0·001) AA: J: β 0·04 (*P* < 0·05) K: β 0·08 (*P* < 0·001) LA: J: β 0·03 (*P* < 0·001) K: β 0·02 (*P* < 0·01)
Markers of inflammation														
IL-1α, IL-1ra, IL-4, IL-8, IL-10, IP-10, TNF-α, MCP-1	Sekikawa A *et al.*, 2010 [44]	Cross-sectional, Japan	100, male, 40–49	Serum	↓ (IL-8) ↔ (IL-1α, IL-1ra, IL-4, IL-10, IP-10, TNF-α, MCP-1)	–	–	–	–	–	–	–	–	Total *n*-3: IL-8: *P*-trend = 0·055
CRP	El-Saed A *et al.*, 2016 [42]	Cross-sectional, Japan	312, male, 40–49	Serum	↔ (EPA + DHA + DPA)	–	–	–	–	↓ (LA + DGLA + GLA + AA+ eicosadienoic acid + DPA)	–	↓	–	Total *n*-6: *r* = –0·19 (*P* = 0·001) LA: *r* = –0·16 (*P* = 0·006)
CRP	Tomiyama H *et al.*, 2011 [40]	Cross-sectional, Japan	2206, male, 44 ± 9	Serum	–	↔	↔	–	–	–	↑	–	↑	AA: β 0·01 (95 % CI 0·00, 0·01), *P* < 0·01 DGLA: β 0·01 (95 % CI 0·00, 0·01), *P* < 0·01
CRP	Kubota Y *et al.*, 2015 [43]	Cross-sectional, Japan	1102, male and female, 40–74	Serum	↓ (ALA + EPA + DHA + DPA) ↓ (EPA + DHA)	↓	↓	–	–	↓ (LA + DGLA + GLA + AA)	↔	↓	↔	Total *n*-3: β –0·089 (*P* < 0·01) EPA + DHA: β –0·091 (*P* < 0·01) EPA: β –0·071 (*P* = 0·03) DHA: β –0·068 (*P* = 0·04) Total *n*-6: β –0·169 (*P* < 0·01) LA: β –0·159 (*P* < 0·01)
Markers of haemostasis and thrombosis														
Plasma fibrinogen	Hassen LJ *et al.*, 2012 [45]	Cross-sectional, Japan	302, male, 40–49	Serum	↓ (EPA + DHA + DPA)	–	–	–	–	–	–	–	–	Total *n*-3: β –0·11 (*P* = 0·041)
PAI-1	Lee S *et al.*, 2012 [46]	Cross-sectional, Japan	313, male, 40–49	Serum	–	–	–	–	–	↓	↓	↓	–	Total *n*-6: β –0·12 (*P* < 0·001) AA: β –0·10 (*P* < 0·001) LA: β –0·04 (*P* < 0·001)
Imaging-based biomarkers														
CAC score	Sekikawa A *et al.*, 2019 [47]	Cross-sectional, Japan	1086, male, 40–79	Serum	↔ (EPA + DHA + DPA)	↔	↓	–	–	–	–	–	–	DHA: OR 0·84 (95 % CI 0·74, 0·94), *P* = 0·03
AoCaS	Mahajan H *et al.*, 2019 [48]	Cross-sectional, Japan	310, male, 40–49	Serum	↔ (EPA + DHA + DPA)	↔	↔	–	–	–	–	–	–	–
CDS	Sekikawa A *et al.*, 2019 [47]	Cross-sectional, Japan	1086, male, 40–79	Serum	↔ (EPA + DHA + DPA)	↔	↔	–	–	–	–	–	–	–
Carotid IMT	Sekikawa A *et al.*, 2011 [49]	Cross-sectional, Japan	310, male, 40–49	Serum	–	↔	↓	–	↔	–	↔	↔	–	DHA: *P*-trend 0·014
Carotid IMT	Dai XW *et al.*, 2014 [50]	Cross-sectional, China	847, male and female, 40–65	Erythrocyte	↔	↔	↓	–	↓	–	–	–	–	DHA: OR 0·58 (95 % CI 0·34, 0·97), *P*-trend 0·037 ALA: OR 0·39 (95 % CI 0·23, 0·67), *P*-trend < 0·001

↑, positive association; ↓, inverse association; ↔, no association; –, not examined; DGLA, dihomo-*γ*-linolenic acid; CAD, coronary artery disease; MI, myocardial infarction; LA, linoleic acid; AF, atrial fibrillation; SBP, systolic blood pressure; DBP, diastolic blood pressure; TC, total cholesterol; FG, fasting glucose; FSI, fasting serum insulin; HOMA-IR, homeostatic model assessment of insulin resistance; AA, arachidonic acid; ALA,*α*-linolenic acid; WC, waist circumference; J, Japanese; K, Korean; IL-1ra, IL-1 receptor agonist; IP-10, inducible protein-10; MCP-1, monocyte chemoattractant protein-1; CRP, C-reactive protein; PAI-1, plasminogen activator inhibitor-1; CAC, coronary artery calcification; AoCaS, aortic calcification score; CDS, CAC density score; IMT, intima media thickness.

#### PUFA biomarkers and CVD events

A prospective cohort study (median 9·6 years) from Taiwan by Chien *et al.* observed no association between plasma EPA, DHA and a composite of CVD events, which included non-fatal MI, fatal CHD, hospitalisation due to percutaneous coronary intervention or coronary bypass surgery and stroke^(28)^. For coronary artery disease, which included MI, angina pectoris and cardiac death, Chei *et al.* found no associations with serum *n*-3 PUFA in a prospective case–control study^(29)^. However, total *n*-6 and LA were inversely associated with coronary artery disease^(29)^.

Sun L *et al.* found inverted–*U* shape curve associations of erythrocyte EPA with total stroke and ischaemic stroke with a threshold at EPA level of 0·70 %^(30)^. A higher level of erythrocyte DGLA was associated with a higher risk of total stroke and ischaemic stroke^(30)^. However, LA was inversely associated with ischaemic stroke and positively associated with intracerebral haemorrhage^(30)^.

For atrial fibrillation, a case–control study from Japan showed EPA was marginally positively associated, but DHA and AA were not associated^(31)^. For total (fatal + non-fatal) MI, two prospective case–control studies from Singapore observed inverse associations with plasma total *n*-3 PUFA, EPA and DHA^(32,33)^. On the contrary, a prospective case–control study from a Japanese cohort, which is characterised by very high *n*-3 PUFA intakes, found no associations between plasma total *n*-3 PUFA, EPA, DHA, DPA and MI^(34)^. No association was found between ALA and MI^(33)^. Analysis of specific *n*-6 PUFA showed that AA was positively associated with MI, but no association was found between LA and MI^(32)^.

In general, there appeared to be an inverse association between total *n*-3, EPA and DHA and MI, but not in populations characterised by very high *n*-3 PUFA intakes. Inconclusive associations between *n*-3 PUFA and other CVD events were observed. Also, inconclusive associations between *n*-6 PUFA and CVD events in Asian populations were observed.

#### PUFA biomarkers and traditional risk factors

For blood pressure, two cross-sectional studies from China (average age 57 years old) reported an inverse association with total *n*-3 PUFA^(35,36)^. However, a smaller cross-sectional study with younger Singaporean participants (∼39 years old) found no association between plasma total *n*-3 PUFA and blood pressure^(37)^. Furthermore, Huang *et al.* found no associations between specific *n*-3 PUFA, EPA, DHA, DPA and blood pressure^(35)^. In a prospective study, Zeng *et al.* reported a marginal inverse association between erythrocyte total *n*-3 PUFA and blood pressure over a mean period of 3·09 years^(36)^. Also, prospectively, EPA, DHA and DPA were found to be inversely associated with blood pressure, while ALA was not associated with blood pressure^(36)^.

In relation to *n*-6 PUFA, total *n*-6 PUFA were found to be inversely associated with blood pressure in a cross-sectional study of Chinese participants from China^(36)^. However, in two other smaller cross-sectional studies, no associations were found^(35,37)^. Furthermore, Huang *et al.* reported no associations between specific *n*-6 PUFA, AA and LA and blood pressure^(35)^. Prospectively, there were no associations between total *n*-6 PUFA and blood pressure^(36)^. In general, there appeared to be an inverse association between total *n*-3 PUFA and blood pressure, while mixed results were found for total *n*-6 PUFA and blood pressure. However, it is recognised that the number of studies available is limited and is mostly cross-sectional in design, which means that the direction of causality cannot be inferred.

For TC, the cross-sectional study from Singapore found an inverse association with plasma total *n*-3 PUFA and EPA^(37)^. Similarly, a cross-sectional study from India found that serum total *n*-3 PUFA (EPA + DHA) was inversely associated with TC and TC:HDL-cholesterol ratio^(38)^. Only the Singapore study examined TC and LDL-cholesterol with plasma *n*-6 PUFA and found that plasma total *n*-6 PUFA and LA were inversely associated with TC^(37)^. For LDL-cholesterol, the study from Singapore found inverse associations with plasma total *n*-6 PUFA, LA and AA^(37)^. For TAG, the cross-sectional study from Singapore found an inverse association with EPA, total *n*-6 PUFA and LA^(37)^. A similar inverse association was found between EPA + DHA, LA and TAG in the study from India^(38)^. Furthermore, plasma total *n*-6 PUFA and LA were positively associated with HDL-cholesterol in the study from Singapore^(37)^.

Only one cross-sectional study in Singapore investigated the association of plasma total *n*-3 and *n*-6 PUFA with fasting blood glucose, fasting serum insulin and homeostatic model assessment for insulin resistance, but no associations were observed^(37)^.

In general, there appeared to be a beneficial effect of EPA + DHA and EPA on TC and TAG. A similar beneficial effect was also observed for total *n*-6 PUFA, especially LA, on blood lipids.

#### PUFA biomarkers and physical signs

For aortic/arterial stiffness, a cross-sectional study in Korean middle-aged males found inverse associations with serum total *n*-3 PUFA and EPA, whereas no association was found with DHA^(39)^. Another larger study in Japanese middle-aged males found no association between serum total *n*-3 PUFA, EPA and DHA and arterial stiffness^(40)^. As for specific *n*-6 PUFA, AA and DGLA were found to be positively associated with arterial stiffness^(40)^.

For obesity, the cross-sectional study from India reported an inverse association between serum ALA and BMI^(38)^. In a cross-sectional study from Singapore, no association was found between plasma total *n*-3 PUFA and both waist circumference and fat mass, but for total *n*-6 PUFA, an inverse association was found with waist circumference but not fat mass^(37)^. In general, findings were mixed, and the limited studies found no consistent associations between PUFA biomarkers and aortic stiffness and obesity in Asian populations.

#### PUFA biomarkers and non-traditional lipid markers

Only one cross-sectional study examined associations between serum *n*-6 PUFA and VLDL-cholesterol, LDL-cholesterol and HDL-cholesterol subclasses, and this involved Japanese and Korean males aged 40–49^(41)^. Total *n*-6 PUFA, AA and LA showed an inverse association with total, large and medium VLDL-cholesterol particle concentrations and a positive association with small VLDL-cholesterol particle concentrations. A similar inverse association was also observed with total and small LDL-cholesterol particle concentrations and a positive association with large LDL-cholesterol particle concentrations. In contrast, total *n*-6 PUFA and LA showed a positive association with large HDL-cholesterol particle concentrations and an inverse association with medium and small HDL-cholesterol particle concentrations. Associations between *n*-3 PUFA and lipoprotein subclasses were not examined.

#### PUFA biomarkers and markers of inflammation

Three cross-sectional studies from Japan investigated the associations between *n*-3 and *n*-6 PUFA and CRP^(40,42,43)^. Only one study from Japan reported an inverse association between total *n*-3 PUFA, EPA, DHA and CRP in 40–74 years old male and female participants^(43)^. In contrast, no associations between total *n*-3 PUFA, EPA, DHA and CRP were reported in two other studies with relatively younger male participants^(40,42)^. Two studies reported an inverse association between serum total *n*-6 PUFA, LA and CRP in both males and females^(42,43)^.

Only one cross-sectional study from Japan examined proinflammatory cytokines and found an inverse association between total *n*-3 PUFA and IL-8^(44)^. No associations were found with the other proinflammatory cytokines.

In general, total *n*-3 PUFA (EPA and DHA), individual EPA and DHA were found to have an inverse association with CRP in older populations. Also, total *n*-3 PUFA were inversely associated with IL-8. Additionally, total *n*-6 PUFA (LA, DGLA, γ-linolenic acid and AA) and LA were found to have an inverse association with CRP.

#### PUFA biomarkers and markers of haemostasis and thrombosis

Two cross-sectional studies examined the association between serum *n*-3 or *n*-6 PUFA and biomarkers related to blood clotting in Japanese males aged 40–49^(45,46)^. One study observed inverse associations between total *n*-3 PUFA (EPA + DHA + DPA) and plasma fibrinogen^(45)^. In the other study, similar inverse associations were observed between total *n*-6 PUFA, AA and LA and plasma PAI-1^(46)^.

#### PUFA biomarkers and non-invasive imaging-based biomarkers

In Japanese, total *n*-3 PUFA and EPA were not associated with CAC score, CAC density score^(47)^ and aortic calcification score^(48)^. Analysis of the DHA level showed an inverse association with the CAC score^(47)^, but no association was found between the CAC density score^(47)^ and aortic calcification score^(48)^.

For carotid IMT, no associations were found with total *n*-3 PUFA and EPA, but an inverse association was found with DHA^(49,50)^. ALA was inversely associated with carotid IMT in the Chinese population from China^(50)^, but not in the Japanese population^(49)^. Analysis of specific *n*-6 PUFA showed that AA and LA were not associated with carotid IMT in the Japanese^(49)^. In general, no associations were found between total *n*-3 PUFA, EPA and non-invasive imaging-based biomarkers. Some associations were observed for DHA and circulating *n*-6 PUFA biomarkers with imaging-based biomarkers, but findings were inconsistent, and studies were limited.

### Risk of bias assessment

All sixteen cross-sectional studies were assessed to be of fair quality, mainly due to poor scoring under the selection domain, where sample size justification and reporting of non-respondents were lacking despite a representative sample and validated ascertainment of exposures (Table 3). Comparability and outcome domain scores were graded with good scores as potential confounders were controlled, assessments of outcomes were objective and appropriate statistical tests and reporting were conducted.

**Table 3.**
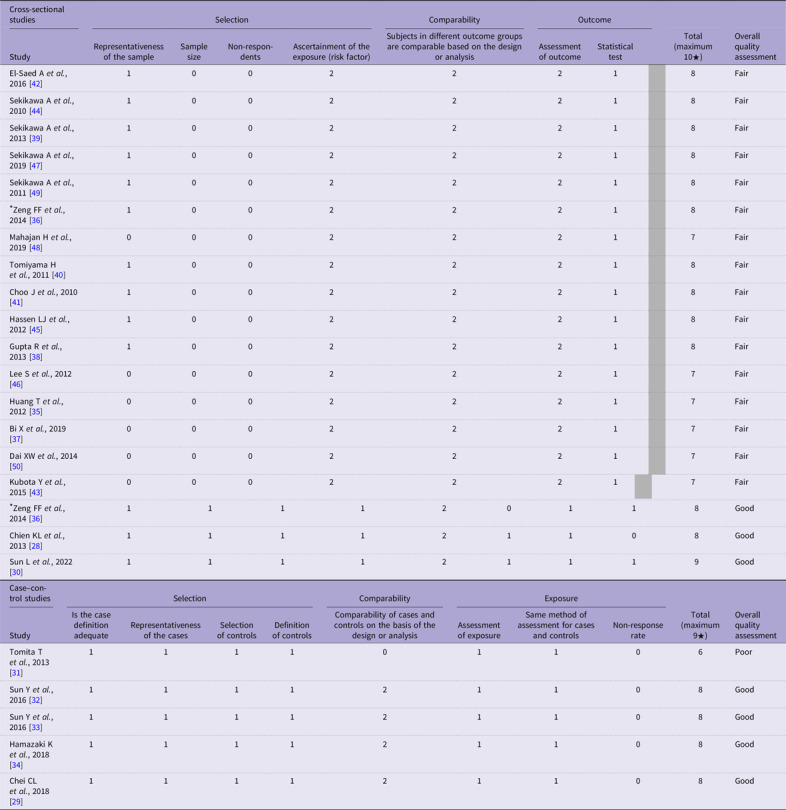
Quality assessment of included studies using the Newcastle–Ottawa Scale

*This study consists of a cross-sectional and prospective cohort component.

The three cohort studies were assessed to be good quality, with good scores across the selection, comparability and outcome domains (Table 3). The selection was representative of the exposed cohort, the non-exposed cohort was drawn from the same community as the exposed, ascertainment of exposure was through the secure record and the outcome of interest was not present at the start of study. Potential confounders were controlled. Outcome assessment was either independent or blind or through record linkage/clinical record, and follow-up was assessed to be long enough.

Four out of five case–control studies were assessed to be of good quality (Table 3). The remaining case–control study was assessed to be of poor quality mainly due to poor comparability from the lack of information on confounders that were adjusted for. Selection domain scores were graded well as case definitions were adequate, cases were representative, community controls were used and controls had no history of outcomes. For the exposure domain, although ascertainment of exposures was through secure records and the same method of ascertainment was used for cases and controls, all five studies did not report on the non-response rate.

## Discussion

We performed a systematic literature review on the associations between *n*-3, *n*-6 PUFA biomarkers and cardiovascular risk factors and events in healthy Asian populations. We observed higher circulating total *n*-3 PUFA to be associated with lower hypertension risk and specifically EPA and DHA to be associated with lower MI risk, reduction in TAG and inflammation. Higher circulating LA was associated with improved lipid profiles and lower inflammation. No consistent associations were observed for circulating *n*-3 and *n*-6 PUFA biomarkers with arterial stiffness, obesity, thrombosis and imaging-based biomarkers. Associations between circulating *n*-6 PUFA biomarkers and CVD events and blood pressure were inconclusive. To our knowledge, this is the first study to review the associations between circulating *n*-3 and *n*-6 PUFA biomarkers and cardiovascular risk factors and events specifically in healthy Asian populations.

This review suggested that total plasma *n*-3, EPA and DHA lowered the risk of total (fatal + non-fatal) MI in Asian populations. This finding is in line with a meta-analysis of nineteen cohort studies mainly from Western countries, which examined the relationship between EPA or DHA concentrations (such as plasma, serum, erythrocytes or adipose tissue) in healthy adults at the start of the study and the risk of developing CHD, including MI^(51)^. Upper quintiles of EPA and DHA were associated with a lower risk of non-fatal MI (quintile 5 *v*. quintile 1 comparison: RR, 0·71 (95 % CI, 0·56, 0·90) *v*. 0·87 (95 % CI, 0·78, 0·97))^(51)^. Supplementation studies also found positive effects of *n*-3 PUFA on MI as summarised in meta-analyses of randomised controlled trial (RCT)^(52–55)^. It is noted that the high circulating *n*-3 PUFA levels in Japanese may reach the plateau level where the associations between circulating *n*-3 PUFA and the risk of MI cannot be detected. One challenge with CVD events is that they are collected in a variety of ways, depending on the study. Further large cohort studies with larger variability of exposure and high-quality trials in Asian populations are required to evaluate the detailed effects of circulating *n*-3 PUFA on specific CVD events.

As for *n*-6 PUFA, only one study found plasma AA to be positively associated with MI in this review. This corroborates with an Israel case–control study, which found that adipose tissue AA was positively associated with acute MI^(56)^. Another Danish case–control study also found a positive association between adipose tissue AA levels and MI^(57)^. AA, derived from the intake of animal foods (e.g. meat, egg and fish), is one of the major dietary long-chain *n*-6 PUFA. One possible mechanism is that AA and its metabolite thromboxane A2 and prostaglandins may stimulate the formation and secretion of testosterone, which is increasingly recognised as related to MI and heart failure in men^(58)^. Further research is warranted to elucidate the effects of AA on MI in Asian populations.

Only one study examined the associations between *n*-3 PUFA and stroke risks in Asian populations. Interestingly, this study found a threshold at the EPA level of 0·70 % by the restricted cubic splines analysis, with inverse associations observed with total stroke and ischaemic stroke above this level. Given the low consumption of marine *n*-3 PUFA among Chinese and the large variation in *n*-3 PUFA levels in Asian populations^(59)^, this would reflect the importance to identify an ideal EPA level for cardio-metabolic health. Evidence linking circulating levels of *n*-3 PUFA with stroke risk in Asian populations is very limited, with only one study found. In Asian populations who had higher stroke rates and substantially different dietary patterns compared with Western populations^(60)^, further larger studies are needed to elucidate these associations.

A beneficial association between total *n*-3 PUFA and blood pressure was found in Asian populations. This is in line with two prospective nested case–control studies in Western populations, which found that circulating total *n*-3 PUFA were inversely associated with the incidence of elevated blood pressure^(61,62)^. This is also supported by a recent umbrella meta-analysis in 2022, which reported a significant inverse relationship between *n*-3 PUFA supplementation and both systolic blood pressure and diastolic blood pressure^(63)^. Subgroup analysis showed that the inverse relationship holds true for healthy populations^(63)^, though the relationship was more significant in subgroups at higher risk of CVD^(64,65)^. Similarly, a meta-analysis of RCT showed that supplementation with EPA significantly reduced systolic blood pressure, while supplementation with DHA significantly reduced diastolic blood pressure^(64)^. It is possible that *n*-3 PUFA improve large artery elasticity through their effects of lowering blood pressure via vasodilation, for example, by enhancing nitric oxide production or release^(66)^. Furthermore, the vasodilatory effects of *n*-3 PUFA on vascular smooth muscle cells are mediated via the opening of ion channels, such as calcium-activated potassium channels and ATP-sensitive potassium channels, resulting in hyperpolarisation and relaxation^(66)^. With regard to the associations between specific circulating *n*-3 PUFA and blood pressure in Asian populations, there is presently no current convincing supporting evidence, and more studies are warranted.

This review suggested circulating *n*-3 PUFA, specifically EPA and DHA, can significantly reduce blood TAG levels in Asian populations. There is a lack of comparable observational studies examining the associations between circulating *n*-3 PUFA and TAG levels in healthy Western populations. However, our findings are in line with RCT, which found that EPA or DHA supplementation (> 2 g/d) lowered TAG concentration, with DHA having a greater TAG-lowering effect^(67)^. The American Heart Association recommended that 4 g/d of *n*-3 PUFA, specifically EPA and DHA together or EPA alone, is clinically useful as monotherapy or in addition to other therapies to reduce TAG^(68)^. Possible mechanisms related to a reduction in plasma TAG by EPA and DHA could be through reduced hepatic lipogenesis and VLDL-cholesterol production and increased chylomicron lipolysis^(69)^.

This review found an inverse association between circulating LA and TAG, TC and LDL-cholesterol and a positive association with HDL-cholesterol in Asian populations. Also, serum LA was found to be inversely associated with large VLDL-cholesterol, VLDL-cholesterol size, total and small LDL-cholesterol particle concentrations and positively associated with large HDL-cholesterol particle concentration and HDL-cholesterol size. In line with our findings, in an observational study from Finland, serum total *n*-6 PUFA and LA were inversely correlated with TAG and VLDL-cholesterol particle size^(70)^. A meta-analysis of RCT found a significant reduction in TC and LDL-cholesterol in studies that employed LA supplementation compared with SFA and monounsaturated fatty acid supplementation^(71)^. Similarly, a systematic review of RCT evaluated the effects of replacing 1 % of energy from SFA with *n*-6 PUFA, predominantly LA, and the results indicated reductions in TAG and LDL-cholesterol^(72)^. Some biochemical studies have been performed and suggested several pathways through which LA might exert its cholesterol-lowering actions. *n*-6 PUFA have been shown to reduce hepatic lipogenesis and concurrently activate lipid catabolism *in vitro*, likely via an inhibition of the activity of sterol regulatory element binding protein-1 in the liver^(73)^.

Inverse associations between total serum *n*-3 (EPA and DHA), individual EPA and DHA and CRP were found in an older Asian population (mean age 58 years old), but no associations in a younger Asian population were found. Similarly, in an epidemiological study of 1123 healthy Italian older subjects (mean age 68·2 years old), higher plasma total *n*-3 PUFA were independently associated with lower levels of proinflammatory markers (IL-6, IL-1 receptor agonist, TNFα, CRP) and higher levels of anti-inflammatory markers (soluble IL-6r, IL-10, TGF-β) independent of confounders^(74)^. Another cross-sectional study in older Australian men and women (mean age 77·6 years old) demonstrated a negative association between erythrocyte membrane EPA and DHA and CRP^(75)^. A meta-analysis provided consistent evidence that marine-derived *n*-3 PUFA supplementation had a significant lowering effect on fasting blood levels of CRP, IL-6 and TNF-α in healthy subjects^(4)^. These studies lend further support for the protective role of *n*-3 PUFA against systematic inflammation in healthy older populations.

Regarding the associations between circulating *n*-6 PUFA and CRP, the findings from this review in Asian populations supported the anti-inflammatory effect of *n*-6 PUFA, especially LA, in Western populations. This is in line with a cross-sectional study that suggested that both serum total *n*-6 PUFA and LA, the predominant *n*-6 PUFA, were associated with lower CRP in Finnish men^(76)^. Conversely, no relationship was found between plasma concentrations of LA and eight markers of inflammation: IL-6, soluble IL-6 receptor, IL-1β, IL-1 receptor antagonist, TNF-α, IL-10, TGF-β and CRP in an epidemiological study of 1123 Italian subjects^(74)^. However, interestingly, those subjects in the lowest quartile of plasma LA concentration had the highest proinflammatory IL-6 and CRP concentrations and the lowest anti-inflammatory IL-10 and TGF-β concentrations^(74)^. Contrary to popular belief, current evidence from RCT and observational studies in healthy human adults have found that increased intake of LA does not increase the concentrations of many inflammatory markers^(77,78)^. Indeed, epidemiological studies have even suggested that LA may be linked to reduced inflammation, though further analysis, specifically in Asian populations, is needed.

One study from our review suggested that total *n*-3 PUFA and EPA, but not DHA, has favourable effects on arterial stiffness^(39)^. Past research that also measured arterial stiffness via carotid-femoral pulse wave velocity, the gold standard measurement for arterial stiffness^(79)^, had shown inverse associations of plasma *n*-3 PUFA^(80,81)^ or *n*-3 supplementation^(82,83)^ with arterial stiffness, in line with the findings in Koreans from this review^(39)^. It is noted that the study from Japan in this review^(40)^ used brachial-ankle pulse wave velocity. The difference in the method of outcome measurement could be the reason that this paper from Japan did not find any association between EPA, DHA and arterial stiffness. One study from our review suggested that AA contributed to arterial stiffness^(40)^. However, a larger US prospective cohort study did not find a significant correlation between AA levels in erythrocytes with arterial stiffness^(80)^. Another prospective cohort study in older adults in Iceland also did not find an association between plasma AA and arterial stiffness^(84)^. The association between *n*-3, *n*-6 PUFA and arterial stiffness appeared inconclusive and further studies are warranted.

In this review, total *n*-3 PUFA were found to be inversely associated with plasma fibrinogen. Fibrinogen plays an important role in the formation of fibrin, which stabilises blood clots. High plasma fibrinogen levels are associated with increased blood clots and, in turn, increased risk for MI^(85,86)^. A meta-analysis of fifteen RCT in humans has confirmed that *n*-3 PUFA supplementation inhibits platelet aggregation^(7)^. This is generally in line with findings that *n*-3 PUFA reduce coagulation and thrombosis^(85)^. Regarding *n*-6 PUFA, the inverse associations between serum total *n*-6 PUFA, AA, LA and plasma PAI-1 found in this review were inconsistent with past studies, which found a positive association between AA and PAI-1^(87)^ and no association between LA and PAI-1.^(88)^. A decrease in plasma PAI-1 could lead to a decrease in blocking of tissue-plasminogen activator, leading to increased clot breakdown and eventually reducing blood clots^(89)^. Further research on the associations between PUFA biomarkers and markers of thrombosis in Asian populations is necessary to verify the current findings.

This review suggested that DHA is protective against atherosclerosis through mechanisms reflected by carotid IMT and CAC score^(47,49,50)^, whereas total *n*-3 PUFA and EPA were not associated with any of the non-invasive imaging-based markers, such as CAC score, CAC density score, aortic calcification score and carotid IMT^(47,49,50)^. This may suggest that DHA may be more anti-atherogenic than EPA. However, no comparable studies exist that examined the association between DHA and the various non-invasive imaging-based atherosclerosis markers. More research is needed to ascertain the relationship between circulating individual PUFA and the various non-invasive imaging-based biomarkers in healthy populations.

This systematic review has several strengths. To our knowledge, this is the first systematic review on the association of *n*-3 and *n*-6 biomarkers with cardiovascular events and risk factors in healthy Asian populations. This review focused on PUFA biomarkers that provided objective measures of different individual *n*-3 and *n*-6 PUFA. The review was performed in accordance with the PRISMA guidelines, and the protocol was registered in PROSPERO. A literature search was done comprehensively in four scientific databases and supplemented by hand searching. Some limitations are worth noting. Due to the heterogeneity of the limited studies identified, a meta-analysis could not be performed. Nonetheless, this review can be valuable in understanding the latest research that has been done in this target population and where future research needs to focus on. Second, as only a limited number of studies for each cardiovascular risk factor/event were identified, findings on a particular outcome may not be generalisable to all Asian populations, and readers should approach the analysis of our findings with caution. Third, there is a lack of studies examining the associations between total and individual *n*-6 PUFA and cardiovascular risk factors and events in healthy Asian populations, resulting in inconclusive associations. Fourth, the high circulating *n*-3 PUFA levels in certain populations, such as the Japanese, may reach the plateau level where the associations between circulating *n*-3 PUFA and risk of any CVD events cannot be detected. Fifth, most of the studies included used cross-sectional design; hence, the possibility of reverse-causality bias could not be eliminated. Last, as a limitation of observational studies, residual confounding cannot be ruled out despite adjusting for various known confounders.

### Recommendations for future research

For studies that examined *n*-3 PUFA, the majority focused on total *n*-3, EPA and DHA. Despite DPA and ALA being present in smaller amounts in the human diet, studies have suggested that DPA and ALA have beneficial effects on CVD^(81,90–93)^. Hence, there could be value for future studies to also examine the role of DPA and ALA on CVD. Future studies should focus more on *n*-6 PUFA and their associations with CVD outcomes. Also, this review highlights the need for more longitudinal studies examining the long-term associations between circulating PUFA and CVD risk factors in Asian populations. It was observed that the definitions of ‘total *n*-3 PUFA’ used in studies in this review were not standardised. While some studies included only EPA and DHA, others also included DPA, ALA or other fatty acids or did not explicitly clarify the composition. Future studies should clearly define the composition of ‘total *n*-3 PUFA’ to ensure consistency and comparability across studies. This applies to ‘total *n*-6 PUFA’ as well. Selective reporting bias was found in several studies, where non-significant findings could only be found in data tables or supplementary info. It is proposed that studies at least briefly report non-significant findings in the main paper to avoid giving the false impression that certain exposures were not studied, when in fact no significant associations were found. Future studies should compare the actual levels of PUFA biomarkers composition in different blood components (whole blood, serum, plasma or erythrocyte) or adipose tissue in Asian populations with those from comparable studies in Western populations when more studies become available. Quality assessment using the NOS revealed that higher quality research papers are needed. Areas to take note in future studies include sample size justification and reporting of non-response rate.

### Conclusion

Overall, we demonstrated that, similar to studies from the Western populations, higher circulating total *n*-3 PUFA were associated with lower hypertension risk, and specifically, EPA and DHA were associated with lower MI risk, reduction in TAG and inflammation. Higher circulating LA was associated with improved lipid profiles and lower inflammation. While significant results were observed for PUFA biomarkers (exposures) and cardiovascular risk factors and events (outcomes) in Asian populations, data were maximumly from three studies for each outcome contributing to the narrative synthesis, and therefore, results should be interpreted with caution. More high-quality and prospective studies on this topic in Asian populations are warranted.

## Footnotes

1 One study consisted of both a cross-sectional and prospective cohort component.
